# The Immune Mind: Linking Dietary Patterns, Microbiota, and Psychological Health

**DOI:** 10.3390/nu18010096

**Published:** 2025-12-27

**Authors:** Giuseppe Marano, Gianandrea Traversi, Osvaldo Mazza, Emanuele Caroppo, Esmeralda Capristo, Eleonora Gaetani, Marianna Mazza

**Affiliations:** 1 Department of Neuroscience, Head-Neck and Chest, Section of Psychiatry, Fondazione Policlinico Universitario Agostino Gemelli IRCCS, Largo Agostino Gemelli 8, 00168 Rome, Italy; 2 Department of Neuroscience, Section of Psychiatry, Università Cattolica del Sacro Cuore, 00168 Rome, Italy; 3 Unit of Medical Genetics, Department of Laboratory Medicine, Ospedale Isola Tiberina-Gemelli Isola, 00186 Rome, Italy; gianandrea.traversi@gmail.com; 4 Spine Surgery Department, Bambino Gesù Children’s Hospital IRCCS, 00168 Rome, Italy; osvaldo.mazza1973@hotmail.it; 5 Department of Mental Health, Local Health Authority ASL Roma 2, 00159 Rome, Italy; 6 Department of Translational Medicine and Surgery, Fondazione Policlinico Universitario A. Gemelli IRCCS, Università Cattolica del Sacro Cuore, 00168 Rome, Italy; 7 Unit of Internal Medicine, Cristo Re Hospital, 00167 Rome, Italy

**Keywords:** gut–brain axis, Mediterranean diet, psychobiotics, probiotics, ultra-processed food, depression, anxiety, short-chain fatty acids, kynurenine, inflammation

## Abstract

**Background/Objectives:** Nutritional patterns influence the gut–brain axis and immune signaling with potential consequences for depression and anxiety. We conducted a review focused on clinically meaningful psychiatric outcomes (symptom severity/diagnosis) to synthesize recent evidence (2020–2025) on Mediterranean-style dietary interventions; ultra-processed food (UPF) exposure; and psychobiotic/prebiotic strategies, integrating mechanistic insights relevant to practice. **Methods:** Searches in PubMed/MEDLINE, Scopus, and Web of Science (January 2020–October 2025) combined terms for diet, Mediterranean diet (MD), UPF, microbiota, probiotics, psychobiotics, depression, and anxiety. Eligible designs were randomized/controlled trials (RCTs), prospective cohorts, and systematic reviews/meta-analyses reporting clinical psychiatric outcomes in adults. We prioritized high-quality quantitative syntheses and recent RCTs; data were extracted into a prespecified matrix and synthesized narratively. **Results:** Recent systematic reviews/meta-analyses support that MD interventions reduce depressive symptoms in adults with major or subthreshold depression, although large, long-term, multicenter RCTs remain a gap. Exposure to UPF is consistently associated with higher risk of common mental disorders and depressive outcomes in large prospective cohorts. Psychobiotics (specific probiotic strains and prebiotics) show small-to-moderate benefits on depressive symptoms across clinical and nonclinical samples, with heterogeneity in strains, dosing, and duration. Mechanistic reviews implicate microbiota-derived metabolites (short-chain fatty acids) and immune–inflammatory signaling (including tryptophan–kynurenine pathways) as plausible mediators. **Conclusions:** Clinically, emphasizing Mediterranean-style dietary patterns, reducing UPF intake, and considering targeted psychobiotics may complement standard psychiatric care for depression. Future work should prioritize adequately powered, longer RCTs with standardized dietary protocols and microbiome-informed stratification to clarify responders and mechanisms.

## 1. Introduction

Depression and anxiety are among the leading causes of global disability, contributing substantially to the burden of disease and loss of quality of life worldwide. Although pharmacotherapy and psychotherapy remain the cornerstones of treatment, increasing attention has been directed toward lifestyle-based interventions as adjuncts to conventional psychiatric care [1,2]. Among these, diet has emerged as a modifiable determinant of mental health, exerting influence through multiple biological pathways involving inflammation, oxidative stress, neurotransmitter metabolism, and the gut–brain axis [3,4].

The gut microbiota–brain axis represents a dynamic bidirectional communication network linking the intestinal environment to the central nervous system via neural, immune, and endocrine signaling [5,6]. This interface plays a pivotal role in regulating immune tone and neuroinflammation, processes known to underlie affective and anxiety disorders. Dysbiosis, an imbalance in microbial composition, can enhance intestinal permeability, activate systemic inflammatory cascades, and alter neurotransmitter synthesis, thereby contributing to depressive symptomatology [7,8].

Over the past five years, clinical research has increasingly focused on three interrelated domains with therapeutic potential for mental health: Mediterranean-style dietary interventions, which emphasize fruits, vegetables, legumes, whole grains, and omega-3-rich fats; ultra-processed food (UPF) exposure, a growing public-health concern linked to adverse metabolic and psychiatric outcomes; and psychobiotic and prebiotic strategies, designed to modulate the gut microbiota and influence neuroimmune pathways.

The Mediterranean diet (MD) has consistently been associated with reduced depressive symptoms and improved psychological well-being. Its anti-inflammatory and antioxidant properties, driven by high intake of polyphenols, fiber, and unsaturated fatty acids, are thought to attenuate neuroinflammatory processes and support neuroplasticity [9]. Recent meta-analyses have demonstrated that structured MD interventions can yield clinically meaningful improvements in depressive outcomes, particularly when adherence is reinforced by dietitian support or digital behavioral tools [10,11]. Recent evidence indicates that Mediterranean-style dietary patterns enhance microbial diversity, increase short-chain fatty acid (SCFA)-producing taxa, and attenuate pro-inflammatory signaling, thereby influencing neuroimmune pathways relevant to mood regulation [11].

Conversely, high consumption of UPF has been linked to poorer psychological outcomes, including elevated risk of depression and anxiety. UPFs, characterized by refined carbohydrates, artificial additives, and low nutrient density, may trigger chronic low-grade inflammation, disrupt microbial diversity, and destabilize glycemic control, all of which contribute to affective dysregulation [12,13]. A 2024 umbrella review aggregating data from large prospective cohorts confirmed a robust, dose–response association between UPF intake and risk of common mental disorders [12].

The third and rapidly expanding area of inquiry concerns psychobiotics, specific probiotics and prebiotics that beneficially affect mental health by modulating gut microbiota composition and function. Randomized controlled trials (RCTs) and meta-analyses have reported small-to-moderate reductions in depressive and anxiety symptoms following supplementation with Lactobacillus and Bifidobacterium strains, particularly in individuals with subthreshold depression or elevated stress reactivity [14,15]. Mechanistically, psychobiotics may exert effects through production of SCFAs, notably acetate, propionate, and butyrate, which enhance gut barrier integrity, regulate microglial activity, and influence gene expression via histone deacetylase inhibition [16]. Parallel evidence implicates the tryptophan–kynurenine pathway, whereby inflammatory activation diverts tryptophan metabolism away from serotonin synthesis toward neuroactive metabolites, linking immune dysregulation to mood alterations [17].

Together, these findings converge on the concept of an “immune mind”, a model in which nutritional patterns shape neurobiological resilience through modulation of the gut microbiota and immune–inflammatory signaling [18,19,20]. Integrating dietary assessment into psychiatric evaluation offers a low-cost, safe, and potentially synergistic avenue for improving treatment outcomes. Yet, the clinical applicability of these insights depends on the consistency, quality, and generalizability of existing evidence.

This review therefore aims to critically evaluate and synthesize recent (2020–2025) clinical studies and meta-analyses addressing: the efficacy of Mediterranean-style dietary interventions in depressive and anxiety disorders; the association between UPF exposure and adverse psychiatric outcomes; and the therapeutic potential of psychobiotic and prebiotic approaches. By integrating mechanistic insights with clinical evidence, this review seeks to provide psychiatrists and nutrition researchers with practical recommendations for the translation of nutritional psychiatry into everyday clinical practice.

## 2. Materials and Methods

### 2.1. Protocol and Reporting

This review was conducted and reported in accordance with the PRISMA 2020 guidelines for systematic reviews [21]. The study aimed to systematically synthesize clinical evidence published between January 2020 and October 2025 investigating the relationship between dietary patterns, microbiota-targeted interventions, and mental health outcomes-specifically depression and anxiety-in adult populations. Given the expected heterogeneity of interventions, populations, and outcome measures, no quantitative meta-analysis was planned. Instead, a structured qualitative synthesis was conducted, emphasizing consistency, methodological quality, and clinical applicability of the available evidence.

### 2.2. Eligibility Criteria

Studies were selected according to predefined inclusion and exclusion criteria formulated using the PICOS framework (Population, Intervention/Exposure, Comparator, Outcomes, Study Design). Population: Adults aged 18 years or older, from general or clinical populations, assessed for depressive or anxiety outcomes using validated diagnostic instruments or standardized rating scales. Interventions/Exposures: MD interventions or adherence-based analyses; UPF exposure or consumption; and psychobiotic strategies including probiotic or prebiotic supplementation. Comparators: usual diet, minimal intervention, lower exposure, or placebo. Outcomes: primary outcomes included change in depressive symptoms, incidence of depression, or remission rates; and secondary outcomes encompassed anxiety symptom changes or incident anxiety disorders when co-reported. Study design: RCTs, quasi-experimental studies, prospective cohort studies, and systematic reviews/meta-analyses (SR/MAs); studies focusing exclusively on pediatric samples, psychosis, cognitive decline, or without validated psychiatric measures were excluded.

Time frame: publications between 1 January 2020 and 22 October 2025. Language: English only.

### 2.3. Information Sources and Search Strategy

A comprehensive literature search was conducted in PubMed/MEDLINE, Scopus, and Web of Science (Core Collection). The search strategy combined MeSH and free-text terms relating to diet and microbiota (“Mediterranean diet,” “dietary pattern,” “ultra-processed food,” “probiotic,” “prebiotic,” “psychobiotic,” “gut microbiota,” “gut–brain axis”) with psychiatric terms (“depression,” “depressive disorder,” “anxiety,” “anxiety disorder”). The final PubMed query was: (“Mediterranean diet”[Mesh] OR “Mediterranean diet”[tiab] OR “dietary pattern”[tiab] OR “ultra-processed food”[tiab] OR “probiotic”[Mesh] OR “prebiotic”[Mesh] OR “psychobiotic”[tiab] OR “gut microbiota”[Mesh] OR “gut-brain axis”[tiab]) AND (“depression”[Mesh] OR “depressive disorder”[tiab] OR “anxiety”[Mesh] OR “anxiety disorder”[tiab]).

Equivalent search strings were adapted for Scopus and Web of Science. Filters were applied for human studies, English language, and publication years 2020–2025. In addition, reference lists of included systematic reviews and key mechanistic papers were manually screened to identify relevant studies not captured by electronic searches.

### 2.4. Study Selection

The screening and selection process was performed in two independent stages by two reviewers (G.M. and M.M.). In the first stage, titles and abstracts were screened for relevance. In the second stage, full texts of potentially eligible articles were retrieved and assessed against inclusion criteria. Discrepancies were resolved by consensus or consultation with a third reviewer (G.T.). Risk of bias for included randomized trials was assessed using the Cochrane risk-of-bias framework, evaluating random sequence generation, allocation concealment, blinding, incomplete outcome data, selective reporting, and funding sources. For observational studies, risk of bias was assessed based on confounding control, exposure/outcome assessment, attrition, and statistical adjustment. Discrepancies between reviewers were resolved through consensus.

**Figure 1:**
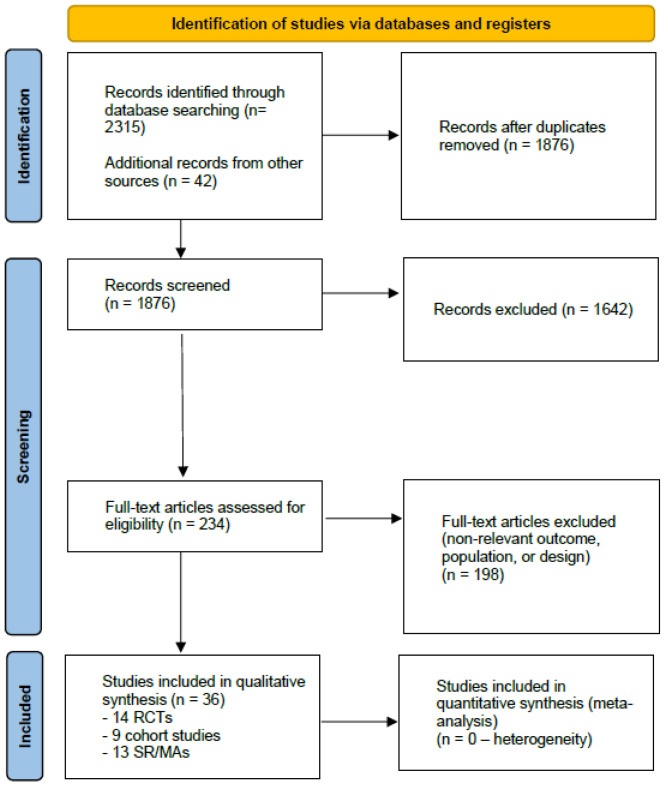
PRISMA 2020 Flow Diagram. Abbreviations. RCT: Randomized Controlled Trial; SR/MA: Systematic Review/Meta-Analysis; UPF: Ultra-Processed Food; MD: Mediterranean Diet.

The process is illustrated in Figure 1 (PRISMA 2020 Flow Diagram). A total of 2357 records were identified (2315 from databases and 42 from reference lists). After removal of duplicates, 1876 records were screened by title and abstract. Of these, 1642 were excluded for irrelevance or lack of psychiatric outcomes. 234 full-text articles were evaluated for eligibility, and 198 were excluded because they did not meet inclusion criteria (wrong population, design, or outcome). Finally, 36 studies were included in the qualitative synthesis, comprising 14 RCTs, 9 cohort studies, and 13 systematic reviews/meta-analyses. No quantitative synthesis was performed due to heterogeneity of interventions, study designs, and outcome measures.

### 2.5. Data Extraction and Synthesis

Data were extracted into a predesigned matrix covering study design, population characteristics, intervention or exposure details, duration, psychiatric outcomes, principal findings, and quality assessment. Extraction was performed independently by two reviewers. Because of substantial methodological and conceptual heterogeneity, particularly regarding dietary definitions, psychobiotic strain variability, and outcome reporting, a narrative synthesis was undertaken. Findings were grouped by thematic domain (MD interventions, UPF exposure, psychobiotic/prebiotic strategies, and mechanistic insights), and consistency and clinical relevance were graded across bodies of evidence.

## 3. Results

### 3.1. Mediterranean-Style Diet (MD) Interventions and Depressive Outcomes

Over the last five years, multiple RCTs and MAs have consolidated the role of the MD as a promising adjunctive intervention for depressive symptoms. Recent studies demonstrate significant reductions in depressive symptom severity compared with control diets or usual care conditions, underscoring the feasibility and clinical potential of MD-based interventions in both clinical and subclinical depression [10,22].

In a comprehensive meta-analysis including over 2000 participants across 14 RCTs, Bizzozero-Peroni et al. reported that adherence to a Mediterranean dietary pattern significantly improved depressive symptoms compared with control diets, with standardized mean differences ranging from −0.22 to −0.40 depending on intervention duration and adherence level [10]. Importantly, these benefits were observed across diverse populations, including individuals with major depressive disorder (MDD), those with subthreshold symptoms, and adults at metabolic risk. The overall certainty of evidence was rated as moderate, suggesting a robust, clinically meaningful association between MD adherence and mood improvement.

These findings were corroborated by Lassale et al. [23], who identified consistent antidepressant effects of MD interventions across both primary prevention and clinical treatment studies, emphasizing that high-fidelity implementation and sustained adherence are critical determinants of efficacy. Interventions incorporating dietitian-led counseling, structured meal planning, or behavioral adherence tools, such as digital prompts, cooking workshops, or group support, tended to achieve greater reductions in depressive scores than those relying solely on written dietary guidance [24].

From a mechanistic standpoint, the MD exerts its antidepressant effects through several complementary pathways. The diet’s high content of omega-3 fatty acids, polyphenols, and dietary fiber reduces systemic inflammation and oxidative stress, thereby modulating neuroinflammatory cascades implicated in depression [25]. Improved gut microbiota diversity associated with MD adherence enhances SCFA production, which strengthens intestinal barrier integrity and influences neurotransmitter metabolism [9,26]. Additionally, MD adherence has been linked to reduced activation of the tryptophan–kynurenine pathway, leading to improved serotonin availability and reduced neurotoxic metabolite production [27].

Despite these encouraging results, several limitations temper the current evidence base. Most available RCTs enrolled small to medium-sized samples (*n* < 250) and were conducted over relatively short durations (typically 8–12 weeks). Moreover, heterogeneity in dietary adherence monitoring and control conditions, ranging from minimal advice to fully structured interventions, complicates direct comparisons. Few studies employed blinded assessors or standardized psychiatric diagnostic tools, which may introduce bias. Nevertheless, the direction and consistency of effects across diverse settings reinforce the clinical relevance and safety of the MD as an adjunctive strategy for depression management [28].

Implementation science insights suggest that adherence strategies play a decisive role in sustaining benefits over time. Interventions supported by personalized meal plans, cooking skill development, and digital adherence tracking yield larger and more durable improvements in depressive symptoms. Conversely, programs with low engagement or poor adherence monitoring often demonstrate attenuated or non-significant effects.

Overall, current research supports the Mediterranean-style diet as a feasible, low-risk, and biologically plausible adjunct in psychiatric care pathways. It is supported by preliminary but promising evidence as a feasible, low-risk adjunct preventive strategy for mental health, particularly in preventive and therapeutic frameworks for depression. Large-scale, long-term RCTs with rigorous adherence assessment and mechanistic endpoints are warranted to confirm durability and generalizability of these effects.

### 3.2. Ultra-Processed Food (UPF) and Common Mental Disorders

Growing epidemiological evidence indicates that high consumption of UPFs, industrial formulations characterized by refined carbohydrates, added sugars, saturated fats, and numerous cosmetic additives, is associated with adverse mental health outcomes, including higher prevalence and incidence of depression and anxiety disorders [12,29]. Over the past five years, several large-scale cohort studies and umbrella reviews have strengthened the link between UPF intake and affective disorders, revealing a robust and biologically plausible dose–response relationship [30].

In a landmark umbrella review encompassing 45 meta-analyses and over 10 million participants, Lane et al. confirmed that high UPF consumption is consistently associated with an increased risk of depression and anxiety symptoms across diverse populations [29]. The association remained significant after adjustment for BMI, socioeconomic factors, and overall diet quality, suggesting an effect independent of total caloric intake.

Mechanistically, the deleterious impact of UPFs on mental health appears multifactorial. Diets high in UPFs promote chronic low-grade inflammation, oxidative stress, and gut microbiota dysbiosis, all of which contribute to neuroimmune dysregulation and affective vulnerability [31]. Excessive intake of emulsifiers, artificial sweeteners, and preservatives alters microbial composition, leading to decreased production of beneficial SCFAs and increased intestinal permeability, which in turn may activate systemic inflammatory pathways and cytokine cascades implicated in depression [26,32]. In parallel, high-glycemic-load foods provoke postprandial glucose spikes and insulin resistance, disrupting energy metabolism and neuroplastic signaling within limbic structures involved in mood regulation.

Recent neuroimaging data suggest that chronic exposure to UPFs may affect reward-related brain circuits, potentially through dopaminergic sensitization and impaired satiety signaling. This neurobehavioral dysregulation may partially explain the bidirectional relationship between emotional eating and depressive symptoms, particularly among individuals with stress-related vulnerabilities or obesity [33].

The magnitude of association between UPF intake and depression risk is comparable to that observed for other modifiable risk factors such as physical inactivity or heavy alcohol use, underscoring the potential public-health relevance of dietary composition beyond caloric balance. Nevertheless, residual confounding remains a concern: individuals with higher UPF consumption often exhibit lower socioeconomic status, higher rates of smoking, and reduced access to health-promoting resources [34]. Despite these limitations, the consistency of findings across diverse cohorts, analytic methods, and geographical regions strengthens the causal inference that UPF exposure negatively influences mental health.

Furthermore, emerging data indicate that reductions in UPF consumption may lead to short-term improvements in psychological well-being and perceived stress, independent of weight change or macronutrient intake. Interventional trials, though still scarce, suggest that replacing UPFs with minimally processed alternatives rich in fiber, polyphenols, and unsaturated fats can improve mood and fatigue within 2–4 weeks, possibly via microbiota restoration and decreased systemic inflammation [35].

These findings suggest that UPF consumption is a modifiable, population-level risk factor for depression and anxiety. Addressing UPF exposure through dietary counseling, food-labeling policies, and public-health campaigns could represent a scalable strategy for primary and secondary prevention of common mental disorders. Future research should integrate dietary assessment with biological endpoints, such as inflammatory cytokines, SCFAs, and gut microbiota composition, to further elucidate the causal pathways linking UPFs and affective outcomes.

### 3.3. Psychobiotics (Probiotics/Prebiotics) for Depression/Anxiety

Over the past decade, and particularly since 2020, the concept of psychobiotics has gained substantial traction in nutritional psychiatry. The term refers to live microorganisms (probiotics) or non-digestible substrates (prebiotics) that confer mental health benefits via modulation of the gut microbiota–brain axis [13,14]. Growing evidence from RCTs and meta-analyses suggests that psychobiotic and prebiotic interventions can attenuate depressive and anxiety symptoms, albeit with modest and variable effect sizes [36,37].

A comprehensive meta-analysis by including 36 RCTs reported that probiotic supplementation led to a statistically significant reduction in depressive symptoms compared with placebo [36]. The greatest improvements were observed in participants with subclinical depression or elevated depressive scores rather than those with established MDD.

Across these studies, probiotic strains belonging to the genera *Lactobacillus* and *Bifidobacterium*, particularly *L. helveticus* R0052, *L. rhamnosus* GG, and *B. longum* NCC3001, have demonstrated reproducible antidepressant and anxiolytic effects in both clinical and non-clinical populations. These effects are thought to be mediated through several convergent biological mechanisms. Psychobiotics modulate systemic and neuroinflammatory signaling, reducing levels of pro-inflammatory cytokines such as interleukin-6 (IL-6), tumor necrosis factor-α (TNF-α), and C-reactive protein (CRP), which are consistently elevated in depressive states [38]. They enhance intestinal epithelial integrity and increase the production of SCFAs, which exert anti-inflammatory, epigenetic, and neuroprotective effects. These SCFAs influence blood–brain barrier permeability and modulate the activity of microglial cells, thereby affecting neuroinflammatory tone and synaptic plasticity [39].

No consensus exists regarding optimal dosing, duration, or responder characteristics, and strain heterogeneity limits the generalizability of findings. Standardization of strain selection and reporting remains a major unmet need.

Psychobiotic supplementation has been shown to normalize hypothalamic–pituitary–adrenal (HPA) axis hyperactivity, leading to lower cortisol levels and improved stress reactivity. In both human and animal studies, probiotics attenuate stress-induced increases in adrenocorticotropic hormone (ACTH) and corticosterone, suggesting a regulatory influence on central stress pathways [40,41]. Finally, modulation of tryptophan metabolism via the kynurenine pathway may underpin psychobiotics’ antidepressant potential. By dampening inflammation-driven indoleamine-2,3-dioxygenase (IDO) activity, these interventions may preserve tryptophan availability for serotonin synthesis, reducing neurotoxic kynurenine metabolites that have been implicated in depression and fatigue [42].

Despite encouraging results, several limitations persist. Considerable heterogeneity exists across studies in probiotic species, dosages, intervention duration, and outcome measures, complicating cross-trial comparison. Many studies enrolled small samples and used self-reported questionnaires rather than clinician-administered scales, potentially inflating effect sizes. Additionally, few trials have performed strain-specific analyses or mechanistic biomarker assessments, such as microbiota sequencing, SCFA quantification, or cytokine profiling, which are essential to delineate causal pathways. A major limitation across dietary and psychobiotic research is the lack of replication, compounded by substantial heterogeneity in strains, dosages, dietary protocols, and outcome measures.

Emerging evidence also supports prebiotic interventions, particularly fructooligosaccharides (FOS) and galactooligosaccharides (GOS), which selectively stimulate the growth of beneficial *Bifidobacteria* and promote SCFA production [43]. Early trials suggest that prebiotic supplementation can improve emotional regulation and reduce cortisol awakening response in healthy adults, though data in clinical populations remain limited.

Compared with depressive symptoms, findings for anxiety outcomes are more heterogeneous. Some RCTs report modest reductions in state or trait anxiety, whereas others show no significant effect, particularly in populations with established anxiety disorders. Variability in assessment tools and sample characteristics contributes to inconsistent results [15]. Overall, psychobiotic effects on anxiety remain less robust and require targeted trials.

Research indicates that psychobiotic and prebiotic interventions exert small-to-moderate but clinically relevant effects on depressive and anxiety symptoms. They appear to complement traditional psychiatric treatments through anti-inflammatory, neuroendocrine, and microbiota-mediated mechanisms. While not a substitute for established pharmacotherapy or psychotherapy, psychobiotics represent a safe, accessible, and biologically grounded adjunct in the management of mood disorders.

### 3.4. Integrative Mechanistic Overview

Across the reviewed studies, converging evidence highlights a shared set of biological pathways linking diet quality, gut microbiota composition, and affective regulation. Mediterranean-style dietary patterns, UPF exposure, and psychobiotic or prebiotic interventions appear to act on common molecular and neuroimmune mechanisms, albeit in opposite directions. Collectively, these findings reinforce the concept of an immune–metabolic continuum underlying mood disorders, wherein nutritional patterns shape inflammatory tone, microbial metabolites, and neurotransmitter homeostasis.

Dietary modulation of systemic inflammation emerges as a central node connecting all three domains. The MD exerts robust anti-inflammatory and antioxidant effects through high intake of omega-3 polyunsaturated fatty acids, polyphenols, and vitamins C and E, which suppress pro-inflammatory cytokines and reduce oxidative stress. Conversely, UPF-rich diets are associated with increased circulating concentrations of IL-6, tumor necrosis factor-alpha (TNF-α), and CRP, as well as higher oxidative stress biomarkers. Chronic low-grade inflammation can cross the blood–brain barrier, activating microglia and perturbing monoaminergic signaling, ultimately leading to anhedonia, fatigue, and depressive mood. Psychobiotic supplementation, on the other hand, appears to attenuate inflammation by restoring intestinal barrier integrity and inhibiting pro-inflammatory cytokine cascades. Certain *Lactobacillus* and *Bifidobacterium* strains downregulate NF-κB activation and decrease plasma IL-6 and TNF-α concentrations. The resulting reduction in peripheral inflammation may restore serotonergic transmission and improve mood [44].

The gut microbiota represents a critical interface through which diet influences brain function. Adherence to a Mediterranean-style diet increases microbial richness and promotes the growth of *Bifidobacterium* and *Faecalibacterium* species, both major producers of SCFAs such as acetate, propionate, and butyrate. These metabolites enhance epithelial tight-junction integrity, reduce intestinal permeability, and modulate immune responses through inhibition of histone deacetylases (HDACs). SCFAs can cross the blood–brain barrier, where they influence neuroinflammation, synaptic plasticity, and neurogenesis. By contrast, UPFs are poor in fermentable fiber and rich in emulsifiers and artificial sweeteners that disrupt microbial balance and decrease SCFA production. This dysbiotic shift may trigger metabolic endotoxemia and systemic immune activation, further reinforcing depressive pathophysiology. Probiotics directly increase SCFA-producing taxa, while prebiotics (e.g., galacto- and fructooligosaccharides) provide the substrates required for their proliferation. Psychobiotics have been proposed as potential modulators of microbial function, although human microbiome responses to probiotic supplementation are highly variable, and some authors report minimal or no significant compositional changes [45].

Another key mechanism linking diet and mood involves the tryptophan–kynurenine pathway, a metabolic route whereby inflammatory activation diverts tryptophan from serotonin synthesis toward neuroactive kynurenine metabolites. Diets rich in anti-inflammatory nutrients (e.g., omega-3 fatty acids, polyphenols) and gut-derived SCFAs may influence IDO activation, preserving tryptophan availability for serotonin production; however, most supporting evidence derives from preclinical studies, and human data remain preliminary. Conversely, UPF-driven inflammation enhances kynurenine formation, resulting in neurotoxic intermediates such as quinolinic acid that promote excitotoxicity and neurodegeneration [44].

Emerging evidence indicates that probiotic supplementation can modulate tryptophan metabolism by reducing IDO activity and increasing circulating serotonin precursors, thereby counteracting inflammatory diversion within this pathway. Such findings underscore the biochemical convergence between anti-inflammatory dietary patterns and microbiota-targeted therapies in normalizing neurotransmitter homeostasis.

Diet also influences mood through regulation of the HPA axis, the primary neuroendocrine stress pathway. Chronic UPF consumption and metabolic inflammation are associated with hypercortisolemia and impaired negative feedback of cortisol signaling, whereas MD adherence and psychobiotic interventions reduce stress reactivity and normalize cortisol awakening response [46]. These effects are hypothesized to be partially mediated by vagal pathways linking gut microbial metabolites to central autonomic circuits, based primarily on preclinical evidence; direct confirmation in humans remains limited.

Cumulative evidence suggests an emerging immune–metabolic model in which diet may act as a potential trigger and modulator of affective vulnerability, although causal pathways remain insufficiently established. Protective dietary patterns such as the MD promote microbiota diversity, SCFA production, and anti-inflammatory tone, stabilizing neurochemical and neuroendocrine function. Conversely, deleterious dietary exposures, notably UPFs, disrupt these regulatory networks, promoting inflammation, dysbiosis, and HPA-axis overactivation. Psychobiotics and prebiotics, positioned mechanistically between these two poles, provide a biologically plausible but still investigational approach of restoring microbial–immune homeostasis and complementing nutritional and pharmacological interventions in depression and anxiety.

**Figure 2:**
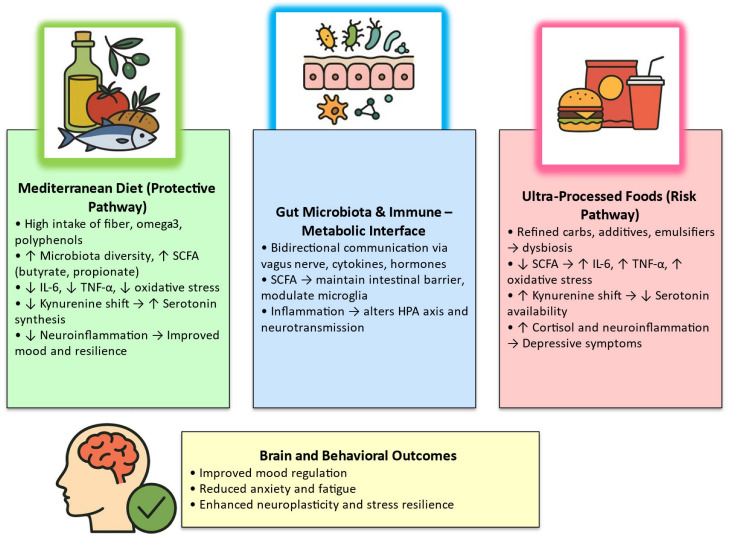
The Immune Mind Model: Integrative Pathways Linking Diet, Microbiota, and Mental Health. Note: The figure illustrates the opposing effects of Mediterranean-style versus ultra-processed dietary patterns on the gut microbiota, immune signaling, and mood regulation. Psychobiotics and prebiotics act as restorative modulators. Abbreviations: SCFA, short-chain fatty acids; IL-6, interleukin-6; TNF-α, tumor necrosis factor-alpha; HPA, hypothalamic–pituitary–adrenal; ↑/↓, increase/decrease.

The interplay among dietary quality, gut microbiota, and immune regulation provides a unifying framework for understanding how nutrition influences the “immune mind.” These mechanistic insights seem to indicate the clinical potential of nutritional psychiatry and justify integrative approaches that combine dietary modification, microbiota-targeted therapy, and lifestyle optimization to contribute to prevent and treat mood disorders. These relationships are summarized in Figure 2, which depicts the bidirectional interactions between diet quality, gut microbiota, immune signaling, and mood regulation, the conceptual core of the “immune mind” model.

Newer prospective syntheses assessing overall diet quality report inverse associations with subsequent depression risk, complementing the interventional MD literature. While residual confounding cannot be excluded, alignment between cohort and RCT/SR evidence strengthens the case for diet-first adjunctive strategies in mood disorders [47].

A concise overview of the principal clinical findings across MD, UPF exposure, and psychobiotic/prebiotic interventions is presented in Table 1.

## 4. Discussion

The relationship between diet, microbiota composition, and mental health has evolved from a theoretical construct to one of the most compelling translational frontiers in psychiatry. Current evidence suggests that the gut is not merely a digestive organ but an immuno-metabolic interface capable of influencing mood and cognition through neural, endocrine, and inflammatory pathways [5,48,49].

Recent randomized trials and meta-analyses indicate that Mediterranean-style dietary interventions can reduce depressive symptoms [1,23,50], that higher consumption of UPF is associated with poorer psychological outcomes [12,33], and that microbiota-targeted strategies such as psychobiotics may exert modest yet clinically relevant benefits on mood and anxiety [51,52,53]. While effect sizes are heterogeneous, the convergence of findings across independent methodologies suggest a link between nutrition, microbiota, and mental health.

Among nutritional models, MD is most consistently linked to favorable psychiatric outcomes. Rich in fiber, omega-3 fatty acids, antioxidants, and polyphenols, the MD attenuates inflammation and oxidative stress, key mechanisms implicated in the pathophysiology of depression [24,28]. Recent meta-analyses have confirmed that structured MD interventions significantly reduce depressive symptom severity, particularly when combined with behavioral support [1,23,50]. The SMILES trial and subsequent RCTs demonstrated that dietary counseling emphasizing whole grains, legumes, fruits, and extra-virgin olive oil improved depressive outcomes independently of weight loss or caloric restriction [54]. The benefits likely arise from metabolic and neurochemical modulation, including enhanced neuroplasticity, altered neurotransmitter synthesis, and reduced systemic inflammation [55]. Adherence remains a pivotal determinant: trials with regular dietitian follow-up or digital adherence support achieved larger symptom reductions than minimal-contact designs [23,56]. This underscores that dietary change in psychiatric care should be conceptualized as a structured behavioral intervention, requiring sustained motivation and clinician guidance.

Conversely, UPF consumption has emerged as a robust risk factor for common mental disorders. UPFs, characterized by refined carbohydrates, trans-fats, additives, and low nutrient density, induce glycemic instability and chronic inflammation. A 2024 umbrella review encompassing over 30 prospective cohorts concluded that higher UPF intake was consistently associated with increased incidence of depression and psychological distress [57]. Though observational, these findings display dose–response relationships and are biologically plausible, given that UPF-rich diets impair microbial diversity, increase intestinal permeability, and elevate circulating pro-inflammatory cytokines [12]. From a clinical standpoint, dietary counseling aimed at reducing UPF represents a pragmatic preventive and adjunctive measure, reinforcing lifestyle recommendations that already target cardiometabolic health.

The field of psychobiotics-probiotics and prebiotics that influence brain function via the gut microbiota—offers a more targeted avenue for intervention. Systematic reviews and meta-analyses published between 2023 and 2025 report small-to-moderate improvements in depressive symptoms following psychobiotic supplementation, particularly with multi-strain formulations containing *Lactobacillus* and *Bifidobacterium* species [14,58]. Effects on anxiety are less consistent but trend positive. Mechanistically, psychobiotics may enhance gut barrier integrity, modulate immune signaling, and influence the synthesis of neuroactive compounds such as γ-aminobutyric acid (GABA) and serotonin [59,60]. However, heterogeneity in strains, dosages, and treatment durations limits comparability across studies. Furthermore, individual differences in baseline microbiota composition and inflammatory status likely moderate therapeutic response [8]. Despite these limitations, psychobiotics are generally safe and well tolerated, justifying their cautious use as adjuncts within integrative psychiatric care, provided that clinicians rely on products supported by high-quality RCTs.

Mechanistic studies further clarify how diet and microbiota affect the central nervous system. SCFAs (acetate, propionate, and butyrate) produced by bacterial fermentation of dietary fiber, regulate microglial activation and gene expression through epigenetic mechanisms [26,61]. Reduced SCFA availability is associated with disrupted gut barrier function and neuroinflammation, phenomena linked to depressive symptomatology [62]. Another pathway of interest is the tryptophan–kynurenine axis: during inflammation, tryptophan metabolism shifts toward kynurenine derivatives, reducing serotonin availability and producing neurotoxic metabolites such as quinolinic acid [63]. Dysbiosis can amplify this shift by promoting systemic inflammation, thereby linking immune activation to mood dysregulation. These mechanisms lend biological plausibility to clinical observations that fiber-rich, anti-inflammatory diets and selected probiotics yield mood benefits.

Collectively, the reviewed evidence suggests that nutrition acts as a modifiable determinant of psychiatric resilience. In contrast to pharmacological treatments, which act downstream on neurotransmitter systems, dietary interventions target upstream processes (immune signaling, oxidative stress, and energy metabolism) that contribute to chronic affective dysregulation [64]. Integrating dietary assessment into psychiatric evaluation may therefore enhance treatment personalization. Collaboration with dietitians and general practitioners can support adherence, while structured tools (e.g., dietary recall or adherence indexes) enable progress monitoring. Nutritional approaches are particularly attractive for their low cost, safety, and broad health benefits, offering a viable complement to psychotherapeutic and pharmacologic interventions.

Despite encouraging results, several methodological challenges remain. Blinding participants to dietary interventions is inherently difficult, raising the possibility of expectancy bias. Adherence assessment varies across studies, ranging from self-report to biomarker validation. For psychobiotics, strain heterogeneity and short intervention durations limit generalizability, while variability in outcome measures complicates synthesis. Observational findings on UPF are susceptible to residual confounding, as UPF consumption correlates with socioeconomic and lifestyle variables such as lower physical activity and poor sleep hygiene [65]. Dietary adherence and intervention feasibility are influenced by cultural norms, food accessibility, and socioeconomic status, which limit generalizability across populations. Publication bias is a concern in nutritional psychiatry, as small positive studies are more likely to be published than null findings. Moreover, industry funding, particularly in probiotic trials, may influence reporting.

Addressing these limitations will require rigorous, multi-center randomized trials with standardized dietary protocols, validated adherence metrics, and long-term follow-up.

Looking forward, multi-omics approaches integrating metagenomics, metabolomics, and immunomics offer exciting opportunities to identify microbial or metabolic signatures predictive of dietary responsiveness [66]. Such data could usher in a new era of precision nutrition in psychiatry, in which interventions are tailored to the individual’s microbial profile and inflammatory state. Early studies already suggest that baseline microbiota composition may predict antidepressant response, hinting at future combined models incorporating diet, microbiota, and pharmacogenomics [67,68]. In this context, nutrition is not merely an adjunct but a potential stratification tool for treatment selection.

From a clinical and public-health perspective, these findings translate into actionable recommendations. Psychiatrists should routinely explore dietary habits as part of holistic assessment, encouraging patterns that emphasize whole foods, plant diversity, and omega-3–rich sources while minimizing UPF [69]. Nutritional education can be integrated into psychoeducation programs, reframing diet as a form of self-care that enhances pharmacologic and psychotherapeutic efficacy. When appropriate, short courses of evidence-based psychobiotics may be proposed, with outcome monitoring through validated instruments such as the PHQ-9 or HADS [70]. At the population level, policies that facilitate access to affordable, minimally processed foods could yield mental-health benefits alongside reductions in metabolic disease burden [71].

Notably, the large MooDFOOD multicenter RCT found no significant preventive effect of multinutrient supplementation or food-related behavioral therapy on depressive outcomes [72]. This null result tempers the generally positive narrative and underscores the complexity of translating nutritional interventions into depression prevention.

## 5. Conclusions

The convergence of clinical, epidemiological, and mechanistic data underscores the reality of the “immune mind”, where nutritional patterns modulate brain function through immune and microbial pathways. MD, characterized by anti-inflammatory and microbiota-friendly properties, appears to confer protection and symptom improvement in depression. Conversely, UPFs promote dysbiosis and systemic inflammation, contributing to psychological vulnerability. Psychobiotics offer a promising, though still developing, adjunctive strategy [73,74].

While mechanistic insights highlight the plausibility of nutritional interventions, their clinical utility remains experimental and adjunctive. Dietary modification and microbiota-targeted strategies may complement standard psychiatric treatments but do not replace established therapeutic modalities.

Future research should prioritize well-designed, longer-term, biomarker-anchored RCTs to clarify mechanisms and identify responders. Bridging nutrition and psychiatry represents not only a scientific imperative but also a clinical opportunity to restore coherence between brain, body, and behavior.

## Figures and Tables

**Table 1:** Summary of recent clinical evidence (2020–2025) linking dietary domains, microbiota, and mental health outcomes.

Domain	Representative Evidence (2020–2025)	Main Findings	Mechanistic Pathways	Clinical Implications
Mediterranean-style diet (MD)	[1,10,23]	↓ depressive symptoms in MDD and subthreshold depression; moderate-quality evidence	Anti-inflammatory, antioxidant, ↑ SCFA, ↓ kynurenine shift	Feasible adjunct to standard psychiatric care; adherence essential
Ultra-processed food (UPF)	[12]	↑ risk of depression and anxiety (25–40% higher risk in top quartile intake)	Dysbiosis, ↑ inflammation, glycemic instability, dopaminergic dysregulation	Reduce UPF consumption; integrate food policy and public-health education
Psychobiotics/Prebiotics	[42]	Small-to-moderate improvement in depressive symptoms (multi-strain, ≥8 weeks)	↑ SCFA, ↓ IL-6/TNF-α, normalized HPA axis, ↓ IDO activation	Safe adjunctive therapy; requires strain-specific standardization

Abbreviations: MD, Mediterranean diet; MDD, Major Depressive Disorder; UPF, ultra-processed food; SCFA, short-chain fatty acids; HPA, hypothalamic–pituitary–adrenal; IDO, indoleamine-2,3-dioxygenase; IL-6, Interleukin-6; TNF-α, Tumor necrosis factor-alpha; ↑/↓, Increase/Decrease.

## Data Availability

No new data were created or analyzed in this study. Data sharing is not applicable to this article.

## Abbreviations

The following abbreviations are used in this manuscript:

ACTH	Adrenocorticotropic hormone
CRP	C-reactive protein
HPA	Hypothalamic–pituitary–adrenal (axis)
IDO	Indoleamine-2,3-dioxygenase
IL	Interleukin
MDD	Major depressive disorder
MD	Mediterranean diet
RCT	Randomized controlled trial
SCFA	Short-chain fatty acids
TNF-α	Tumor necrosis factor-alpha
UPF	Ultra-processed food
